# Sleep Problems in Children With Developmental Delays and Neurodevelopmental Comorbidities: A Retrospective Cross-Sectional Study

**DOI:** 10.7759/cureus.76837

**Published:** 2025-01-03

**Authors:** Kin Lok Wong, Shuk Kuen Chau, So Lun Lee

**Affiliations:** 1 Paediatrics, Queen Elizabeth Hospital, Kowloon, HKG; 2 Paediatrics and Adolescent Medicine, Queen Mary Hospital, Hong Kong, HKG

**Keywords:** attention deficit hyperactivity disorder, autistic spectrum disorder, developmental delay, neurodevelopmental comorbidities, sleep, sleep problems

## Abstract

Background

Sleep disturbances are more common in children with developmental delay (DD) than in typically developing children. There is limited research on whether sleep impact is more pronounced in children with DD and comorbidities.

Objective

Our study compared sleep patterns and disturbances in children with DD and neurological or psychobehavioral comorbidities to those with isolated DD.

Methodology

We conducted a single-center retrospective study utilizing a parent-rated questionnaire on neurodevelopmental clinic attendees over six months. Subjects were categorized into three groups: (1) isolated DD; (2) DD with comorbid neurological conditions; and (3) DD with comorbid psychobehavioral conditions.

Results

A total of 529 subjects were included. The results revealed minimal differences in sleep schedules between the groups. More than half of the sample (276, 52.2%) had insufficient sleep hours, with no significant variation across groups. A substantial proportion of the sample took regular naps, with 340 (79.3%) on weekdays and 279 (69.9%) on weekends.

Compared to the isolated DD group, the comorbid neurological group showed a higher sleep resistance score, while comorbid psychobehavioral conditions were associated with higher sleep resistance scores and lower sleep parasomnia scores. A lower portion of the comorbid neurological group had their parents satisfied with their sleep quality (16, 32.7%, vs. 121, 52.2%). A higher percentage of parents in the comorbid psychobehavioral group reported significant daily life impact due to their children’s sleep problems (65, 28.0%, vs. 41, 17.1%).

Conclusions

The sleep schedule in all three groups closely resembled each other. All three groups exhibited a high prevalence of sleep-related problems. Regular screening for sleep problems is recommended, particularly for children with DD and neurological or psychobehavioral comorbidities.

## Introduction

Sleep disturbance was more prevalent among children with developmental delay (DD) than typically developing children. The prevalence of sleep problems was reported in 40%-80% of children with DD [1-3], compared to 20%-40% of typically developing children [4-6].

In real clinical settings, some patients had isolated DD, while others had comorbid neurological or psychobehavioral diseases. For the latter group of patients, they were well-known for having sleep-related problems. Several studies evaluating sleep problems in children with neurological conditions like cerebral palsy or epilepsy [7-10], psychobehavioral diseases like autistic spectrum disorder (ASD) or attention-deficit/hyperactivity disorder (ADHD) [11-13], and genetic syndromes like Angelman Syndrome [14] have been published. Reported sleep problems included sleep-related breathing disorder, difficulty in falling asleep, short sleep duration, night waking, and parasomnia. While concomitant neurological or psychobehavioral comorbidities in children with DD are commonly encountered in real-world practice, there have been insufficient studies investigating whether children with DD and these comorbidities experience more pronounced disruptions in sleep patterns or the impacts of sleep problems.

Duchess of Kent Children's Hospital (DKCH) is one of the eight centers offering child developmental assessment in Hong Kong, which takes care of many children with DD. While developmental assessment is crucial in these children, their sleep problems are another aspect of care that we need to pay attention to. We aimed to compare the sleep patterns, sleep disturbances, and impacts of sleep problems in children with DD with neurological and psychobehavioral comorbidities and those with isolated DD only.

## Materials and methods

Children with developmental problems were recruited when they attended follow-ups at the neurodevelopmental clinic at DKCH between January 1 and June 30, 2016. Eligible subjects were those aged between two and 12 years. Parents were provided with the questionnaire on sleep upon attendance. Information on bedtime, wake time, daytime nap habits, and sleep problems was collected. Demographic information including sex, age, height, weight, medical comorbidities, use of sedative medications (sedative antihistamines, antiepileptics, and dystonia medication), and use of intranasal steroids and inhalational steroids were obtained from the subject’s electronic medical record. Subjects were excluded if questions on sleep schedules were left unfinished or if more than 40% of other questions on sleep problems were unanswered [15]. Given the estimated total number of children with DD in Hong Kong to be 60,000, with a margin of error of 5% at a 95% confidence level, the required sample size was 382.

Ethics

The study was approved by the Institutional Review Board of the University of Hong Kong/Hong Kong West Cluster Research Ethics Committee of the Hospital Authority of Hong Kong (UW 21-416).

Questionnaire

A screening questionnaire was designed concerning the Children’s Sleep Habits Questionnaire (CHSQ). The screening questionnaire collected information on sleep schedule, sleep problems, and parental satisfaction with children’s sleep. CHSQ, being validated in typically developing children aged 4-10 years, is a retrospective parent-reported questionnaire for the evaluation of sleep behavior [16]. The frequency of sleep-related habits is being scored as either *usually* (5-7 times per week), *sometimes* (2-4 times per week), or *rarely* (0-1 time per week). A score of 1, 2, and 3 is assigned to the answer of *rarely*, *sometimes*, and *usually*, respectively. However, the original version contained 45 items, among which 33 items were scored, making it undesirable as a short screening tool in a busy clinic setting. Relevant questions on domains of sleep resistance, sleep regularity, sleep duration, presence of parasomnia, sleep-disordered breathing, and daytime sleepiness were selected from the CHSQ as a brief screening tool. For more information on the questionnaire designed to collect parental responses, please refer to the Appendix. Parents were instructed to report the child’s sleep habits for the immediate past week.

Categorization of subjects

Recruited subjects were categorized into three groups: (1) isolated DD; (2) DD with comorbid neurological conditions; and (3) DD with comorbid psychobehavioral conditions. We hypothesized that children with DD and neurological or psychobehavioral comorbidities would have more disrupted sleep schedules and would report more sleep problems, compared to isolated children with DD. The primary outcome was the comparison of sleep schedules and the prevalence of sleep problems between the three groups, with data from the group with comorbid neurological or psychobehavioral conditions compared to the reference control group, i.e., the isolated DD group. The secondary outcomes included the bedtime, wake time, nocturnal sleep duration, nap time, and sleep onset delay between the three study groups. Questionnaire subdomain score, parent-rated satisfaction with sleep quality, and sleep problem impact were compared.

Comorbid neurological conditions of interest included cerebral palsy and epilepsy because sleep disturbances had been well reported in these two disease entities [17-19]. Diagnoses of ASD or ADHD were grouped under comorbid psychobehavioral conditions due to current evidence of overlapping features of sleep disturbances between them [20,21]. Since the sleep pattern and requirements for preschoolers (less than six years old) and school-aged children vary, the reported sleep pattern was analyzed separately between the two age groups, and the nocturnal sleep duration was compared to the American Academy of Sleep Medicine (AASM) age-specific recommended sleep duration [22].

Statistical analysis

Categorical demographic data were compared using the chi-square test or Fisher Exact test, whereas continuous variables were compared using the independent t-test or Mann-Whitney U test. Little’s Test of Missingness was performed for entries with less than 40% missing data to confirm missing completely at random (MCAR). Expectation maximization imputation was employed to replace the missing data if MCAR was confirmed [15]. For examining overall group differences, either parametric tests, such as analysis of variance (ANOVA), or non-parametric tests, such as the Kruskal-Wallis test, were utilized based on data distribution characteristics. Following the identification of significant global effects, post hoc pairwise comparisons were conducted with adjustments for multiple testing, such as Bonferroni correction, to mitigate the risk of Type I errors. The means of questionnaire total scores and subdomain scores were compared. Linear regression analysis was used to adjust for and identify important contributors to questionnaire scores that differed significantly between groups. Logistic regression was used to recognize factors that contributed to significant differences in parent-rated sleep quality and sleep problem impact between groups. Confounding factors being incorporated in regression analyses were identified as demographic factors significantly differed between the subgroups. All analyses were conducted by Statistical Package for the Social Sciences version 28 (IBM Corp., Armonk, NY). All analyses with *P*-values <0.05 were considered statistically significant.

## Results

**Figure 1:**
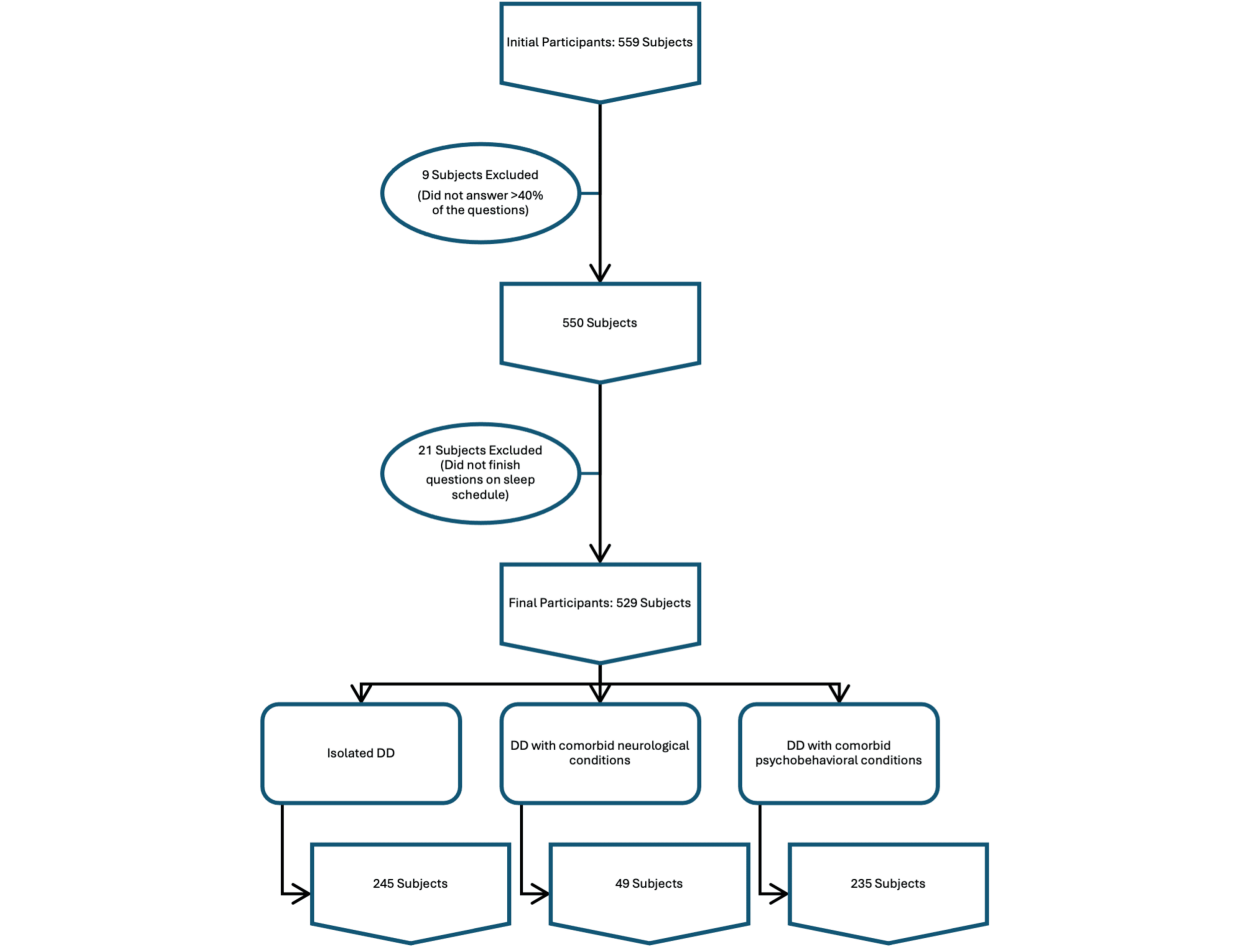
Flowchart of the subject recruitment process. DD, developmental delay

Parents of 559 subjects answered the questionnaires. Nine subjects, who did not answer more than 40% of the questions, were excluded from the analysis. Twenty-one subjects were excluded as parents did not finish all questions on the sleep schedule. Thus, 529 subjects were included in the data analysis (Figure 1). The median age of the subjects was three years. There were 410 (77.5%) subjects aged below six years, i.e., preschool age. The majority, accounting for 380 (71.8%) subjects, were male subjects. The median (interquartile range [IQR]) body mass index (BMI) of the subjects was 15.9 (14.9-17.1) kg/m^2^ (Table 1).

**Table 1:** Participants’ demographic characteristics and medication use. ^a^Comparisons between the DD-only group and the DD plus neurological diseases group. ^b^Comparisons between the DD-only group and the DD plus psychobehavioral diseases group. ^c^Between all groups’ comparison *P*-value. *P-value < 0.05 was considered statistically significant. DD, developmental delay; IQR, interquartile range; BMI, body mass index

Demographics	Overall (*n *= 529)	DD only (*n *= 245)	DD plus neurological diseases (*n *= 49)	*P*-value^a^	DD plus psychobehavioral diseases (*n *= 235)	*P*-value^b^	*P*-value^c^
	Median (IQR)/Number (%)				Median (IQR)/Number (%)		
Age (years)	3.0 (3.0-5.0)	3.0 (3.0-5.0)	5.0 (3.0-8.0)	0.009*	3.0 (2.0-5.0)	>0.999	0.003*
Gender							
Male	380 (71.8%)	151 (61.6%)	31 (63.3%)	0.873	198 (84.3%)	<0.001*	<0.001*
Female	149 (28.2%)	94 (38.4%)	18 (36.7%)		37 (15.7%)		
BMI (kg/m^2^)	15.9 (14.9-17.1)	15.6 (14.7-16.6)	15.5 (14.6-17.0)	>0.999	16.3 (15.3-17.6)	<0.001*	<0.001*
Race							
Asian	504 (95.3%)	233 (95.1%)	47 (95.9%)	-	224 (95.3%)	-	0.062
Caucasians	10 (1.9%)	8 (3.3%)	1 (2.0%)		1 (0.4%)		
Others	15 (2.8%)	4 (1.6%)	1 (2.0%)		10 (4.3%)		
Medication							
Psychostimulants	9 (1.7%)	0 (0.0%)	0 (0.0%)	-	9 (3.8%)	-	-
Antiepileptics	34 (6.4%)	0 (0.0%)	34 (69.4%)		0 (0.0%)		
Dystonia medications	8 (1.5%)	0 (0.0%)	8 (16.3%)		0 (0.0%)		
Sedative antihistamines	17 (3.2%)	8 (3.3%)	2 (4.1%)		7 (3.0%)		
Intranasal steroids	10 (1.9%)	4 (1.6%)	0 (0.0%)		6 (2.6%)		
Inhalational steroids	15 (2.8%)	6 (2.4%)	2 (4.1%)		7 (3.0%)		

There were 245 subjects with isolated DD, 49 subjects with DD and comorbid neurological conditions, and 235 subjects with DD and comorbid psychobehavioral conditions. The majority of the subjects with isolated DD did not take any medication. The use of medicines in the other two subgroups was related to their underlying conditions, including antiepileptic medications and dystonia medications in 34 (69.4%) and 8 (16.3%) of children with comorbid neurological conditions, and psychostimulant medications in 9 (3.8%) children with comorbid psychobehavioral conditions.

Factors including age, gender, weight, and use of sedative medications that significantly differed between the three subgroups were selected as confounding factors in the regression analysis.

Sleep schedule and patterns

Sleep patterns were recorded on weekdays and weekends, respectively (Table 2). The absolute between-group time difference in bedtime, wake time, and nocturnal sleep duration was minimal. The bedtime and wake time on weekends generally lagged half an hour compared to those on weekdays. The median (IQR) nocturnal sleep duration on weekdays and weekends were similar, measuring 9.8 (9.0-10.5) hours and 10.0 (9.5-10.5) hours, respectively. It revealed that 276 (52.2%) subjects of the entire sample had insufficient sleep hours recommended for their age on weekdays. The percentage of subjects with inadequate nocturnal sleep duration was similar among the three subgroups, measuring 126 (51.4%) subjects in isolated DD, 24 (49%) subjects in DD with comorbid neurological conditions, and 126 (53.6%) subjects in DD with comorbid psychobehavioral conditions. The overall sleep pattern was slightly better on weekends.

**Table 2:** Sleep schedules and patterns in developmental delay group and comparison groups. ^a^Comparisons between the DD-only group and the DD plus neurological diseases group. ^b^Comparisons between the DD-only group and the DD plus psychobehavioral diseases group. ^c^Between all groups’ comparison *P*-value. *P-value < 0.05 was considered statistically significant. IQR, interquartile range; DD, developmental delay

Outcome	Overall	DD-only	DD plus neurological diseases	*P*-value^a^	DD plus psychobehavioral diseases	*P*-value^b^	*P*-value^c^
	Median (IQR)/Number (%)	Median (IQR)/Number (%)	Median (IQR)/Number (%)		Median (IQR)/Number (%)		
Weekday							
Bedtime	22:00 (21:30-22:30)	22:00 (21:30-22:30)	21:45 (21:00-22:30)	0.696	22:10 (21:30-23:00)	0.016*	0.002*
Wake time	07:45 (07:00-08:00)	07:40 (07:00-08:00)	07:00 (06:30-07:47)	0.006*	08:00 (07:15-08:30)	0.211	0.001*
Nocturnal sleep duration (hours)	9.8 (9.0-10.5)	10.0 (9.3-10.5)	9.5 (8.9-10.0)	-	9.6 (9.0-10.3)	-	0.124
Sleep insufficiency - Preschooler	243 (88.0%)	114 (90.5%)	17 (70.8%)	-	112 (88.9%)	-	0.812
Sleep insufficiency - School-aged	33 (12.0%)	12 (9.5%)	7 (29.2%)		14 (11.1%)		
Napping - Average duration (hours)	2.0 (1.3-2.0)	2.0 (1.5-2.0)	1.5 (1.0-2.0)	-	2 (1.0-2.0)	-	0.255
Need for napping - Preschooler	309 (89.3%)	150 (88.8%)	22 (95.7%)	-	137 (89.0%)	-	0.924
Need for napping - School-aged	31 (37.3%)	8 (27.6%)	12 (63.2%)		11 (31.4%)		
Weekend							
Bedtime	22:30 (22:00-23:00)	22:30 (22:00-23:00)	22:30 (21:30-23:00)	-	22:30 (22:00-23:00)	-	0.131
Wake time	08:30 (08:00-09:17)	08:30 (08:00-09:00)	08:00 (07:00-09:00)	-	08:30 (08:00-09:30)	-	0.092
Nocturnal sleep duration (hours)	10.0 (9.5-10.5)	10.0 (9.5-10.5)	10.0 (9.4-10.5)	-	10.0 (9.5-10.5)	-	0.121
Sleep insufficiency - Preschooler	187 (95.9%)	84 (94.4%)	14 (87.5%)	-	89 (98.9%)	-	0.749
Sleep insufficiency - School-aged	8 (4.1%)	5 (5.6%)	2 (12.5%)		1 (1.1%)		
Napping - Average duration (hours)	2.0 (1.5-2.0)	2.0 (1.5-2.0)	2.0 (1.0-2.0)	-	2.0 (1.0-2.5)	-	0.233
Need for napping - Preschooler	247 (78.4%)	120 (80.5%)	19 (90.5%)	-	108 (74.5%)	-	0.136
Need for napping – School-aged	32 (38.1%)	10 (34.5%)	12 (63.2%)		10 (27.8%)		
Sleep onset latency							
Less than 20 minutes	283 (53.7%)	136 (56.0%)	30 (61.2%)	-	117 (49.8%)	-	0.163
20-60 minutes	224 (42.5%)	102 (42.0%)	17 (34.7%)		105 (44.7%)		
>60 minutes	20 (3.8%)	5 (2.1%)	2 (4.1%)		13 (5.5%)		

Nap requirements were observed in 340 (79.3%) subjects and 279 (69.9%) subjects on weekdays and weekends, respectively. The median (IQR) nap duration was 2 (1.3-2.0) hours on weekdays and 2 (1.5-2.0) hours on weekends. Similarly, the percentage of nap requirement and nap duration did not differ significantly between the isolated DD control group and neurological or psychobehavioral comorbidities groups. While nap was a typical behavior in preschoolers, our analysis showed that there were still 31 (37.3%) subjects and 32 (38.1%) subjects of school-aged children requiring a nap on weekdays and weekends. Moreover, there was a trend of a higher proportion of school-aged children with comorbid neurological impairment taking a nap than the other two subgroups.

Overall, 224 (46.3%) subjects had a significant sleep onset latency of more than 20 minutes, and 20 (3.8%) subjects took even longer than 60 minutes to reach sleep onset. The pattern of sleep onset latency was similar among the three subgroups.

Sleep problem questionnaire score

Only scores of the sleep resistance and parasomnia subdomains differed significantly between the three subgroups, while the mean total questionnaire scores and scores of other subdomains were similar (Table 3).

**Table 3:** Questionnaire subdomain score in developmental delay group and comparison groups. ^a^Comparisons between the DD-only group and the DD plus neurological diseases group. ^b^Comparisons between the DD only group and the DD plus psychobehavioral diseases group. ^c^Between all groups’ comparison *P*-value. ^*^P-value < 0.05 was considered statistically significant. DD, developmental delay; SD, standard deviation

Subdomain score	Overall	DD-only	DD plus neurological diseases	*P*-value^a^	DD plus psychobehavioral diseases	*P*-value^b^	*P*-value^c^
	Mean ± SD				Mean ± SD		
Sleep resistance (max = 15)	4.66 ± 1.76	4.38 ± 1.54	5.37 ± 2.51	<0.001*	4.81 ± 1.74	0.023*	<0.001*
Sleep regularity (max = 6)	2.79 ± 1.06	2.76 ± 1.01	2.96 ± 1.29	-	2.78 ± 1.07	-	0.485
Sleep duration (max = 9)	6.57 ± 1.02	6.63 ± 1.00	6.39 ± 1.08	-	6.54 ± 1.03	-	0.285
Parasomnia (max = 9)	7.08 ± 1.52	7.28 ± 1.40	6.90 ± 1.71	0.318	6.90 ± 1.59	0.019*	0.016*
Sleep-disordered breathing (max = 9)	6.69 ± 1.12	6.76 ± 1.01	6.86 ± 1.10	-	6.58 ± 1.15	-	0.114
Daytime sleepiness (max = 9)	7.89 ± 1.14	7.98 ± 1.04	7.86 ± 1.30	-	7.82 ± 1.20	-	0.305
Total score (max = 57)	35.68 ± 3.19	35.79 ± 2.93	36.33 ± 3.59	-	35.43 ± 3.35	-	0.158

Compared to the isolated DD group, both DD with comorbid neurological conditions (5.37 ± 2.51 vs. 4.38 ± 1.54, *P *< 0.001) and DD with comorbid psychobehavioral conditions (4.81 ± 1.74 vs. 4.38 ± 1.54, *P *= 0.023) scored higher in the sleep resistance subdomain. After controlling for confounding factors, the presence of comorbid conditions and the use of sedative medications were associated with a higher sleep resistance score, while an age above six years was associated with a lower sleep resistance score (Table 4).

**Table 4:** Linear regression of the questionnaire subdomain score. ^*^P-value < 0.05 was considered statistically significant. DD, developmental delay; *B*, unstandardized coefficient; SE, standard error; β, standardized coefficient

	Dependent variable							
	Sleep resistance score				Parasomnia score			
Independent variable	B	SE	β	*P*-value	B	SE	β	*P*-value
School-age (≥6 years old)	-0.575	0.189	-0.137	0.003*	0.428	0.165	0.117	0.010*
Female	-0.231	0.172	-0.059	0.181	-0.143	0.150	-0.042	0.341
Overweight (BMI ≥ 23.0 kg/m^2^)	-1.261	0.665	-0.082	0.058	1.138	0.580	0.085	0.050
Use of sedative medications	0.728	0.221	0.145	0.001*	-0.446	0.193	-0.103	0.021*
Disease category	0.183	0.081	0.099	0.024*	-0.206	0.071	-0.129	0.004*

In the parasomnia subdomain, DD with comorbid psychobehavioral conditions had a lower mean score than the isolated DD group (6.90 ± 1.59 vs. 7.28 ± 1.40, *P *= 0.019). Regression analysis indicated that the presence of comorbid conditions and the use of sedative medications were associated with a lower parasomnia subdomain score, while an age above six years was associated with a higher parasomnia subdomain score (Table 4).

Parent-rated satisfaction

In comparison with the isolated DD group, a significantly higher percentage of parents in the group of DD with comorbid neurological conditions were dissatisfied with their child’s current sleep quality (33, 67.3%, vs. 104, 42.6%, *P *= 0.002) (Table 5). However, after logistic regression, it showed that the presence of comorbid conditions was not a significant factor. Meanwhile, the use of sedative medications was associated with lower parent satisfaction, whereas more parents showed satisfaction in their child’s sleep quality in subjects older than six years old (Table 6).

**Table 5:** Parent-rated satisfaction on sleep quality and impact of sleep problems in DD group and comparison groups. ^a^Comparisons between the DD-only group and the DD plus neurological diseases group. ^b^Comparisons between the DD-only group and the DD plus psychobehavioral diseases group. ^c^Between all groups’ comparison *P*-value. ^*^P-value < 0.05 was considered statistically significant. DD, developmental delay

	Overall	DD-only	DD plus neurological diseases	*P*-value^a^	DD plus psychobehavioral diseases	*P*-value^b^	*P*-value^c^
	Number (%)				Number (%)		
Satisfaction on current sleep quality							
Dissatisfied	248 (47.2%)	104 (42.6%)	33 (67.3%)	0.002*	111 (47.8%)	0.270	0.006*
Satisfied	277 (52.8%)	140 (57.4%)	16 (32.7%)		121 (52.2%)		
Impact of sleep problems on daily life							
No	405 (77.9%)	199 (82.9%)	39 (81.3%)	0.835	167 (72.0%)	0.006*	0.014*
Yes	115 (22.1%)	41 (17.1%)	9 (18.8%)		65 (28.0%)		

**Table 6:** Binary logistic regression of parent-rated satisfaction on sleep quality and impact of sleep problems. ^*^P-value < 0.05 was considered statistically significant. OR, odds ratio; 95% CI, 95% confidence interval

	Dependent variable			
	Satisfaction on current sleep quality		Sleep problems impact on daily life	
Independent variable	OR (95% CI)	*P*-value	OR (95% CI)	*P*-value
School-age (≥6 years old)	2.212 (1.372-3.566)	0.001*	1.527 (0.924-2.525)	0.099
Female	0.814 (0.541-1.226)	0.326	1.237 (0.758-2.018)	0.395
Overweight (BMI ≥ 23.0 kg/m^2^)	20.868 (0.034-42.323)	0.999	0.448 (0.052-3.899)	0.467
Use of sedative medications	0.347 (0.178-0.676)	0.002*	2.185 (1.092-4.371)	0.027*
Disease category	0.555 (0.244-1.259)	0.159	1.900 (1.196-3.017)	0.007*

Parent-rated impact of sleep problem

A higher percentage of parents in the DD with comorbid psychobehavioral conditions group than isolated DD group complained that the children’s sleep problems affected their daily life greatly (65, 28.0%, vs. 41, 17.1%, *P *= 0.006) (Table 5). Logistic regression also supported that both the presence of comorbid psychobehavioral conditions and the use of sedatives were associated with significantly more parental complaints of children’s sleep problems affecting daily life (Table 6).

## Discussion

Our study did not find children with DD and neurological or psychobehavioral comorbidities having more disrupted sleep schedules than isolated DD. The sleep pattern, nap requirement, and sleep onset latency in children with isolated DD or children with DD and neurological or psychobehavioral comorbidities were similar.

Sleep problems were prevalent in children with DD [1-3,13,18]. Sleep health can significantly affect learning and behavior. Our study was designed to evaluate whether sleep patterns, sleep problems, and their impacts were more pronounced in children with DD and neurological or psychobehavioral comorbidities, as this clinical situation is commonly encountered in real-world practice.

In our study, half of all respondents did not fulfill AASM age-specific sleep hour recommendations [22], among which 243 (88%) subjects were preschoolers, echoing the finding of Tso et al. [23]. Although the prevalence of age-specific sleep insufficiency was not well established locally, Tso et al. [23] showed that only 11% of healthy Chinese preschoolers achieved age-specific sleep requirements in a local survey. In addition, our study showed that the presence of neurological or psychobehavioral comorbidities did not add to the percentage of subjects having inadequate sleep duration.

Nap requirement was reported in nearly all children younger than three years old, less than a third at five years old, and nearly absent in children above seven [24]. However, 340 (79.3%) subjects of all had daytime naps, which was much higher than the reported values. More importantly, there were still 31 (37.3%) of school-aged subjects reporting a need for daytime naps. While difficulty in falling asleep with prolonged sleep onset latency and the aforementioned insufficient sleep hours could be contributing factors, early school schedules and the presence of undiagnosed sleep problems could play a role.

From the sleep questionnaire result, sleep pattern, regularity, and duration were similar among the three groups. These patients shared a common phenomenon of poor sleep habits and sleep deprivation. Our study showed that neurological or psychobehavioral comorbidities would have more sleep problems in the parts of sleep resistance and parasomnia compared to children with isolated DD. Sleep resistance score was higher in patients with DD and comorbid psychobehavioral conditions compared to the control group. Similar results had been reported in previous studies [5,11,13,25], showing that children with psychobehavioral diseases like ASD or ADHD were more resistant to sleep than typically developing or delayed children. It was likely a multifactorial effect. Families of children with psychobehavioral diseases were prone to have inadequate setups for sleep onset [26]. Evening restlessness and poor self-organization led to difficult sleep initiation [13]. Psychoactive medication also played a role in association with increased sleep onset difficulties [25], although only 9 (3.8%) of our psychobehavioral comorbid subjects were on psychostimulants. On the other hand, behavioral sleep problems, especially sleep resistance, sleep initiation, and sleep maintenance, have been well reported in children with neurological disorders [27], which was reproduced in our study result. A lower parent-rated parasomnia score was observed in the group of comorbid psychobehavioral conditions compared to the isolated DD group, yet its clinical importance needed to be evaluated. It was not uncommon that the parents might underreport parasomnia behavior [13,28]. Therefore, parental education on sleep hygiene awareness may benefit this group of patients. After control for confounders, age older than six years was significantly associated with lower sleep resistance scores and higher parasomnia scores. Owens et al. [29] reported the lower occurrence of sleep resistance problems in school-age kids could be related to better coping and stress handling in older age kids. Parasomnia was also more commonly reported in school-aged kids.

Regression analysis showed that the use of sedative medications and age older than six years, but not the presence of neurological comorbidities, were significant factors associated with parental satisfaction with sleep quality. We speculated that medication frequencies, ease of medication preparation, and administration might interrupt children’s sleep schedules and sustainability, intensify parental burden, and affect their satisfaction. Meanwhile, higher parental satisfaction with sleep in subjects older than six years could be related to parental adaptation of children’s sleep behavior with time and fewer nap requirements in school-aged children. On the other hand, more impactful sleep problems in subjects having comorbid psychobehavioral conditions or receiving sedative medications were likely secondary to the increase in non-rapid eye movement (NREM) parasomnia with sedative medications [30] and the cyclic relationship between disturbed sleep and children’s inability to regulate emotion and behavior.

Notably, our study was unique in comparing the group with comorbid neurological or psychobehavioral conditions to the isolated DD group. Future studies using objective measurements, such as actigraphy, could help strengthen the validity of the results.

The interpretation of questionnaire scores in our study was limited due to the lack of validation of the questionnaire design, which weakens the generalizability of our results. Clinicians encounter a broad age range of children presenting with DDs. Thus, including children aged 2-12 years enhances the clinical applicability and relevance of our findings. However, a major limitation on this approach require validity evaluation for the questionnaire responses from children aged 2-3 years and 11-12 years. Additionally, the standard CHSQ was too lengthy to be used as a quick screening tool in clinical settings. Although we developed a concise version of the questionnaire, validating the questionnaire items would enhance the generalizability of our findings for future analyses. A significant limitation of our study was the disparity in group sizes. The number of subjects in the comorbid neurological conditions group was notably lower than in other groups, resulting in baseline heterogeneity. Extending the study period and including more subjects across different groups in future research might help to mitigate this bias. It is crucial to acknowledge that the signs and symptoms of comorbid neurological and psychobehavioural conditions often manifest throughout childhood growth and development. Therefore, it is plausible that children in the younger age group may initially present without defined comorbid conditions, which may become apparent and be diagnosed later in life. This developmental trajectory introduces a potential diagnostic bias. We attempted to reduce this bias by adjusting for the confounding effect of age. Furthermore, a limitation of our study is that data collection occurred in 2016, resulting in a time gap between data collection and the present time. This temporal gap could affect the relevance and applicability of our findings due to advancements in medical practices, diagnostic criteria, and treatment modalities that may have emerged since then.

## Conclusions

Sleep schedules in children with neurological or psychobehavioral comorbidities closely resembled those of children with isolated DD, with both groups sharing the common trait of inadequate sleep duration. A high prevalence of sleep-related problems was observed in all three groups to different degrees. The comorbid neurological group showed a higher sleep resistance score, while a higher parent-rated score in sleep resistance and a lower parasomnia score were reported in the psychobehavioral group children. Overall, a high proportion of subjects had sleep insufficiency, suggesting the need to enhance sleep hygiene and sleep education in these groups of patients. Delayed children, particularly those with neurological or psychobehavioral comorbidities, should be regularly offered screening for sleep problems, which could impact their neurobehavioral functioning and quality of life.

## Appendices

Appendix: Childhood Sleep Quality Index (Parent-report)

What time does your child usually wake up and go to bed? How long is the sleep duration?

1.    Night bedtime:

PM＿＿hour＿＿minute (Weekday)

PM ＿＿hour＿＿minute (Weekend)

2.    Morning Wake-up time:

AM＿＿hour＿＿minute (Weekday)

AM＿＿hour＿＿minute (Weekend)

3.    Typical amount of sleep:

＿＿hour＿＿minute (Weekday)

＿＿hour＿＿minute (Weekend)

4.    Any habit for napping

Start time:pm __ (hr) __ (min) (Weekday)

Total nap time __ (hr) __ (min) (Weekday)

Start time:pm __(Hr)__(min) (Weekend)

Total nap time __(hr) __(min) (Weekend)

How often does the following item occur in your child? Please select the number of times it occurs in a typical week.

                                                                                                               Usually          Sometimes        Rarely

                                                                                                             （5-7）         （2-4）             （0-1）

5.      Go to bed at the same time at night                                           1□                 2□                     3□

6.      Wake up at the same time in the morning                                  1□                 2□                     3□

7.      Sleep too little                                                                               1□                 2□                     3□

8.      Sleep too much                                                                             1□                 2□                     3□

9.      Sleep the right amount                                                                 1□                 2□                     3□

10.    Snort during sleep                                                                         1□                 2□                     3□

11.     Move a lot during sleep                                                                 1□                 2□                     3□

12.    Awaken alarmed by a frightening dream                                      1□                 2□                     3□

13.    Awaken during night                                                                      1□                 2□                     3□

14.    Wake up by him/herself                                                                 1□                 2□                     3□

15.    Wake up in negative mood                                                            1□                 2□                     3□

16.    Take a long time to become alert in the morning                         1□                 2□                     3□

17.    Seem tired after waking up in the morning                                   1□                 2□                     3□

18.    Appear very sleepy or fallen asleep during riding in car              1□                 2□                     3□

19.    How long does it usually take your child to fall asleep on bed?

1□  Less than 20 minutes      2□  20-60 minutes       3□  More than 60 minutes

20.    Do you think your child has the following sleep problems?

                                                              None        A little bit     Quite Severe    Severe     Very Severe

Difficulty falling asleep                             1□              2□              3□              4□              5□

Difficulty staying asleep                           1□              2□              3□              4□              5□

Wake up too early                                     1□              2□              3□              4□              5□

21.    Overall, are you satisfied/dissatisfied about child’s current sleep quality?

1□  Dissatisfied     2□  Satisfied

22.    To what extent do you think your child’s sleep problems affect his/her daily life (e.g. day tiredness, ability to handle schoolwork, concentration, memory, emotions etc)

1□  No impact        2□ Quite much impact

23.    Do you want your child to participate the treatment if we have relevant services in future?

1□  Yes                   2□  No
